# A Chemical Lure for Trapping Both Sexes of *Amata phegea* L.

**DOI:** 10.3390/insects13111051

**Published:** 2022-11-15

**Authors:** Szabolcs Szanyi, Antal Nagy, István Szarukán, Zoltán Varga, Júlia Katalin Jósvai, Miklós Tóth

**Affiliations:** 1 Institute of Plant Protection, Faculty of the Agricultural and Food Sciences and Environmental Management, University of Debrecen, Böszörményi út 138., H-4032 Debrecen, Hungary; 2 Department of Evolutionary Zoology and Human Biology, Faculty of Science and Technology, University of Debrecen, Egyetem tér 1., H-4032 Debrecen, Hungary; 3 Plant Protection Institute, CAR, Herman Otto u. 15., H-1022 Budapest, Hungary

**Keywords:** benzyl acetate, bisexual attractant, eugenol, monitoring, phenylacetaldehyde, trapping

## Abstract

**Simple Summary:**

The development of feeding attractant lures is an actual topic of pest management studies. Compounds present in different flower scents can synergize with each other providing new mixtures appropriate for detecting and monitoring lepidopterans, not only in pests but also in faunistical and ecological studies. The synergizing effect of eugenol and benzyl acetate for phenylacetaldehyde was known for some species of Noctuidae and Pyralidae but in the case of Erebidae, *Amata phagea* is the first species for which it is shown. The lure attracted both sexes and may serve as the basis for further chemo ecological and even ecological studies of the species. Additionally, our data help to reveal the whole effect range of the studied compounds.

**Abstract:**

The addition of synthetic eugenol and benzyl acetate to the known floral chemical and moth attractant phenylacetaldehyde synergized the attraction of *Amata phegea* (Lepidoptera: Amatidae). Traps baited with the ternary blend caught ca. four times more *A*. *phegea* moths than traps baited with phenylacetaldehyde alone. Both female and male moths were attracted; in a preliminary test, the female numbers caught were almost double compared to the males. Most *A. phegea* were caught when the blend was formulated in a dispenser with medium release rates. Traps baited with the ternary lure in polyethylene bag dispensers detected a single well-pronounced peak in seasonal trapping, suggesting that this multicomponent bisexual lure could be efficient enough to be applied to the detection and monitoring of female and male *A. phegea*.

## 1. Introduction

Since pheromone structures for hundreds of pest and non-pest moths are known (www.pherobase.com accessed on 11 January 2022), traps baited with synthetic pheromones are used in a multitude of ways to detect and monitor the occurrence of a high diversity of Lepidoptera. However, sex pheromones of moths attract male specimens only [1]. Bisexual lures would overcome this drawback, by being attractive to both sexes.

Recently, a synthetic bisexual lure has been developed for the silver Y moth (*Autographa gamma* L., Lepidoptera: Noctuidae) [2], which is an important polyphagous pest of vegetables and field crops in Eurasia and North Africa [3]. In the course of field experiments targeting *A. gamma*, we observed frequent sizeable side catches of the non-pest *Amata (Syntomis) phegea* L. (Lepidoptera: Erebidae, Arctiinae, Syntomini) in the traps with various attractant combinations. In this paper, we present detailed trapping results on *A. phegea,* which eventually resulted in the development of a synthetic bisexual lure for attracting the species. *Amata phegea* is widely distributed in Europe with the exception of the northern and mostly Mediterranean parts [4], and above all common in mesophilous forested or scrubby habitats. The larvae are polyphagous and feed mostly on low herbaceous plants, hibernate, and can often be found after hibernation also on the lowest parts of the tree trunks feeding on mosses. The moths are day-flying and have a relatively long activity period from the second half of May to mid-July, depending on geographical latitude and elevation [5]. According to some earlier surveys, *A. phega*, and possibly also other *Amata* spp., are aposematically protected by 2-isopropyl-3-methoxypyrazine and 2-sec-butyl-3-methoxypyrazine [6,7]. This can be interpreted as an example of Müllerian mimicry with the sympatric zygaenid species *Zygaena ephialtes* [8,9]. According to some earlier records, the larvae are also protected by released droplets of a toxic secretion from a specialized structure [10]. Thus, for a more detailed survey of the chemical ecology of this species, it would be necessary to develop effective methods for trapping possibly a large number of specimens.

Phenylacetaldehyde (PHENAL) is recognized as broadly attractive to Lepidoptera [11,12,13,14]. However, its effect is relatively weak and, in most species, captured in traps baited with PHENAL only, is too low and sporadic. So, we concentrated our present studies on the relative importance of the addition of EUG and BENZAC (as possible synergists) on the attraction of *A. phegea*.

## 2. Materials and Methods

### 2.1. Field Tests

Tests aimed to compare moth catches in traps were conducted at several sites in Hungary (detailed in Section 2.4) using generally accepted methods in trapping experiments of the same nature [15].

Traps were arranged as blocks so that each block contained one trap of each treatment. Traps within blocks were separated by 8–10 m, and blocks were sited at least 30 m apart. At each test site, 5 blocks of traps were operated. Traps were inspected at several days’ intervals (preferably twice weekly) when captured insects were recorded and removed.

In the tests, funnel traps CSALOMON^®^ VARL (Plant Protection Institute, CAR, Budapest) were used. These traps have routinely been used for trapping several moth species of larger size [14,16,17]; photos of the trap can be viewed at www.csalomontraps.com (accessed on 28 September 2022). For killing captured insects, a small piece (1 cm × 1 cm^2^) of a household anti-moth insecticide strip (Chemotox^®^ SaraLee, Temana Intl. Ltd., Slouth, UK; active ingredient 15% dichlorvos) was placed into the catch container of traps.

### 2.2. Baits

Synthetic compounds (>95% purity) applied in baits were obtained from Sigma-Aldrich Kft. (Budapest, Hungary). The same dispenser types as in [2] were used, namely:

PE bag dispenser: a 1 cm piece of dental roll (Celluron^®^, Paul Hartmann AG, Heidenheim, Germany) was placed into a tight polyethylene bag made of 0.02 mm linear polyethylene foil. The dimensions of the polyethylene sachets were ca. 1.5 cm × 1.5 cm^2^. The dispenser was attached to a plastic strip (8 cm × 1 cm^2^) for easy handling when assembling the traps. For making up the baits, compounds were administered onto the dental roll and the opening of the polythene bag was heat-sealed while the dispensers were wrapped individually in pieces of aluminum foil. Earlier experience showed that the bait did not lose its efficacy during several weeks of field exposure [14]; hence, we decided that it was safe to replace the lures at 4-week intervals. The dose of single compounds was 100 mg/dispenser. In case of testing mixtures, compounds were loaded into the same dental roll in a single dispenser.

PE vial dispensers: bait dispensers were prepared by adding 100 mg amounts of synthetics into 0.7 ml polyethylene lidded vials (No. 730, Kartell Co., Italy, wall thickness ca 0.5 mm). After loading, the lids of the dispensers were closed, and the dispensers were wrapped individually in pieces of aluminum foil.

PP syringe dispensers: the dispenser consisted of a ca. 4 ml volume polypropylene tube, similar in shape to an injection syringe, which contained a 3 cm piece of dental roll (Celluron^®^, Paul Hartmann AG, Heidenheim, Germany). When preparing the baits, 400 mg of the compounds was administered onto the dental roll through the larger opening at the end of the syringe, then this opening was sealed. At the beginning of the experiment, in the field, the thin tube at the other end of the syringe was cut, and compounds could evaporate and move to the exterior environment through the resulting 4 mm ID hole. 

### 2.3. Statistical Analysis

As is frequently found in field trapping experiments, the catch data (even after transformation) did not fulfill requirements for parametric analysis. Therefore, data were analyzed by the non-parametric Kruskal–Wallis test. When the Kruskal–Wallis test showed significance (*p* = 5%), differences between treatments were analyzed by pairwise comparisons with Mann–Whitney U test. 

All statistical procedures were conducted using the software packages StatView^®^ v4.01 and SuperANOVA^®^ v1.11 (Abacus Concepts, Inc., Berkeley, CA, USA).

### 2.4. Experimental Details

Experiment 1. The objective of the test was to check for the effect of BENZAC and EUG when added singly or together to PHENAL. Treatments included (formulated in PE bag dispensers): PHENAL on its own, binary mixtures of PHENAL with BENZAC or EUG, the ternary combination of PHENAL + BENZAC + EUG and unbaited controls.

The test was run at Agárd, Fejér county, 28 May–12 September 2012. 

Experiment 2. In this test, the performance of the ternary blend of PHENAL + BENZAC + EUG formulated in three different types of dispensers was compared. Treatments included PE vials, PE bags, PP syringe dispensers, and unbaited controls. Although no measurements of release rates were performed, we supposed that PE vial dispensers were emitting at a lower rate than PE bag dispensers (because their wall was much thicker), and PP syringe dispensers were emitting faster than PE bag dispensers, since evaporated compounds could diffuse directly through the open hole.

The test was run parallel at two sites: Szeged, Csongrád county, 1 June–23 October 2012, and Vésztő, Békés county, 1 June–22 October 2012.

## 3. Results

In the present paper, we report only on catches of *A. phegea* in the course of the experiments. A previous report has already been published on catches of the pest noctuids *Autographa gamma* L., which was the original target of the experiments, and of *Macdunnoughia confusa* Steph., (a closely related plusiine moth) [2]. Several other moths were also captured, typical species were the noctuid *Helicoverpa armigera* Hbn., and the pyralids *Nomophila noctuella* Den. et Schiff. and *Ostrinia nubilalis* Hbn. Since none of them showed apparent catch increases over catches with PHENAL alone, we got the impression that their catches were probably due to the presence of PHENAL in the different lures, and thus data on these species are not discussed in detail here.

**Figure 1:**
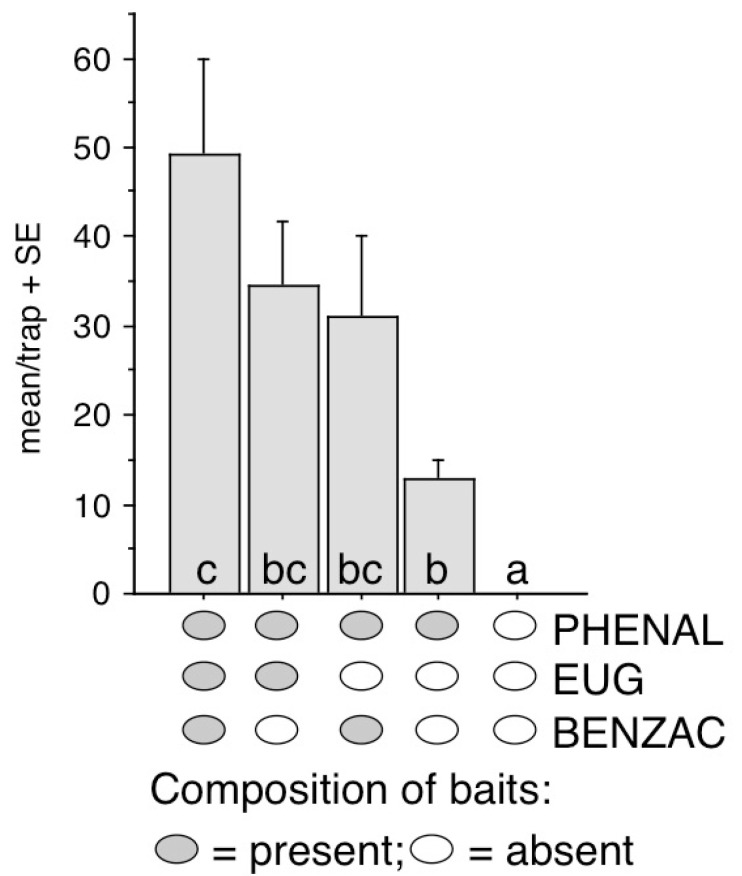
Mean/trap catches of *Amata phegea* in traps baited with phenylacetaldehyde (PHENAL), and its binary and ternary combinations with eugenol (EUG) and benzyl acetate (BENZAC) in Exp. 1 at Agárd. Total caught in test: 637 moths. Columns with same letter are not significantly different at *p* = 5% by Kruskal–Wallis, followed by pairwise comparisons with Mann–Whitney U test.

In Experiment 1, all baited treatments caught significantly more than unbaited traps (Figure 1). The highest mean catches of *A. phegea* were recorded in traps baited with the ternary mixture. The two binary blends showed catches that were numerically lower than, but not significantly different from, the ternary blend. PHENAL alone caught more than controls but was not significantly lower than the two binary blends. The ternary blend caught significantly more than PHENAL alone. 

**Figure 2:**
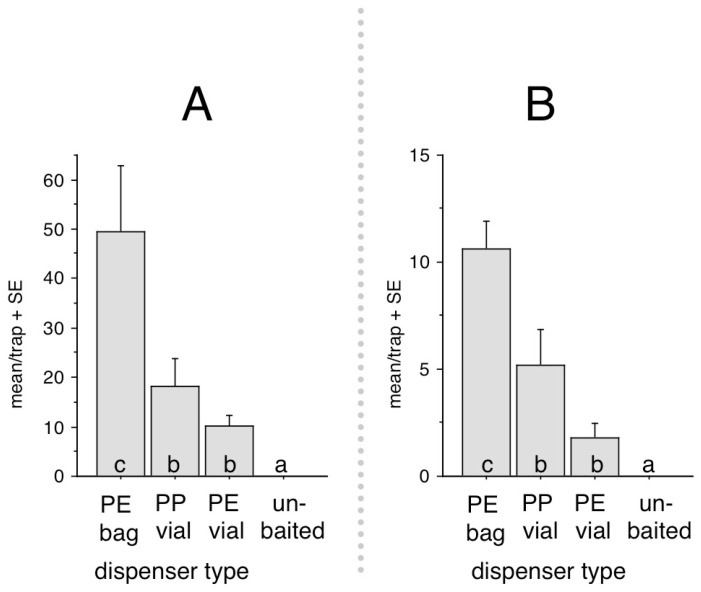
Mean/trap catches of *Amata phegea* in traps baited with the ternary combination of phenylacetaldehyde (PHENAL), eugenol (EUG), and benzyl acetate (BENZAC) in three different types of dispensers in Experiment 2. (**A**) = Szeged, total caught in test: 390 moths. (**B**) = Vésztő, total caught in test: 88 moths. For significance refer to Figure 1.

In Experiment 2, numbers caught showed similar tendencies at both sites, with the ternary blend formulated into PE bag dispensers catching significantly more than any of the other treatments (Figure 2). The other two dispenser types loaded with the ternary blend caught more than unbaited controls, which caught nil.

**Figure 3:**
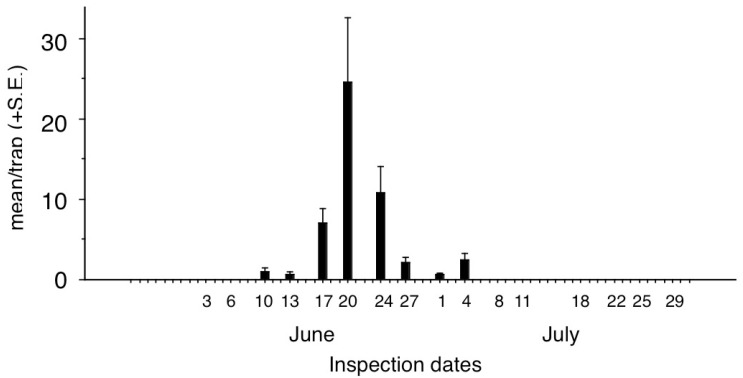
Seasonal distribution of catches of *Amata phegea* in traps baited with the ternary mixture of phenylacetaldehyde (PHENAL), eugenol (EUG), and benzyl acetate (BENZAC) (in PE bag dispensers) at Agárd (data from Experiment 1). Total caught in test: 246 moths.

Seasonal distribution of catches in traps baited with the ternary blend in PE bag dispensers started on 10 June, peaked sharply on 20 June, and the last catches were recorded on 4 July (Figure 3).

## 4. Discussion

Results of the present study clearly show that with the addition of EUG and BENZAC, synergized catches of *A. phegea* over catches to PHENAL alone, thus the optimal lure for this species should contain all three components together. All three compounds are widespread odorants present in the floral scent of many flowers, which may include plant species *A. phegea* adults feed on.

In this respect, *A. phegea* behaved similarly to the noctuids *A. gamma*, *M. confusa*, *Autographa californica* Speyer, and *Xestia c-nigrum* L. [2], which also could be optimally trapped with the same ternary mixture.

PHENAL-containing lures attract many Lepidoptera, mainly noctuids and pyralids (www.pherobase.com accessed on 9 September 2022). However, to the best of our knowledge, this is the first report for the tribe Syntomini (Erebidae). 

There is an increasing number of other cases in the literature where the basic attractive activity of PHENAL alone [11,12] could be increased by the addition of further floral compounds. To mention only a handful of such, the addition of (*β*)-myrcene to PHENAL increased catches of *Pyrausta orphisalis* (Walker) and *Udea profundalis* Packard (Lepidoptera, Crambidae) [18], and the addition of 4-methoxy phenethyl alcohol to PHENAL dramatically elevated catches of *O. nubilalis* [19]. *A. californica* catches were enhanced when PHENAL was presented with *cis*-jasmone or with *β*-myrcene [20,21].

Thus, it appears that the screening of frequently occurring floral odorant compounds can be a viable opportunity in the search for powerful feeding attractant lure combinations for pest or non-pest moths. Such lures would be bisexual attractants, evoking responses from both females and males of the given species. In the case of *A. phegea,* no detailed experiments in this respect have been performed within the framework of the present study, however, in a separate faunistical survey, traps baited with a lure containing PHENAL + EUG + BENZAC caught a total of 51 female and 29 male *A. phegea* (Sz. Szanyi, unpublished). Further in-depth studies should investigate whether sex ratios in the trap captures reflect the actual sex ratios of the given population or not.

Among the dispenser types baited with the ternary lure, *A. phegea* clearly came in the greatest numbers to PE bag; thus, in future trials for detection and monitoring, this dispenser type appears to be most suitable. 

This idea is supported by the results of the seasonal study (Figure 3), where the single well-pronounced peak corresponding to a single adult flight period (beginning between 10 June and 4 July, peaking on 20 June), reflected the known univoltine character of the species [4]. 

For *A. phegea*, a synthetic sex attractant has also been reported as (*Z,Z,Z*)-3,6,9-heneicosatriene [7]. This sex attractant lure attracted only males. Further comparative studies should show the relative efficiency of the sex attractant lure vs. the bisexual lure described in the present study and should inform the situations and research aims to which the lures can be applied.

## Data Availability

Raw data supporting the results in the paper was uploaded into the Zenodo open repository: doi:10.5281/zenodo.7320428; URL: https://zenodo.org/record/7320428#.Y3LoWnaZNPY; CITE: Szanyi Szabolcs, Nagy Antal, Szarukán István, Varga Zoltán, Jósvai Júlia K., & Tóth Miklós. (2022). A chemical lure for trapping both sexes of *Amata phegea* L. https://doi.org/10.5281/zenodo.7320428.
